# New fixation approach for transverse metacarpal neck fracture: a biomechanical study

**DOI:** 10.1186/s13018-018-0890-2

**Published:** 2018-07-25

**Authors:** Yung-Cheng Chiu, Ming-Tzu Tsai, Cheng-En Hsu, Horng-Chaung Hsu, Heng-Li Huang, Jui-Ting Hsu

**Affiliations:** 1 School of Medicine, China Medical University, Taichung, 404 Taiwan; 2 Department of Orthopedic Surgery, China Medical University Hospital, Taichung, 404 Taiwan, Republic of China; 3 Department of Biomedical Engineering, Hungkuang University, Taichung, 433 Taiwan; 4 Department of Orthopaedics, Taichung Veterans General Hospital, Taichung, 407 Taiwan; 5 Sports Recreation and Health Management Continuing Studies-Bachelor’s Degree Completion Program, Tunghai University, Taichung, 407 Taiwan; 6 School of Dentistry, College of Dentistry, China Medical University, 91 Hsueh-Shih Road, Taichung, 40402 Taiwan; 7 Department of Bioinformatics and Medical Engineering, Asia University, Taichung, 413 Taiwan

**Keywords:** Fifth metacarpal neck fracture, Bone plate, K-wire, Cerclage wire

## Abstract

**Background:**

Fifth metacarpal neck fracture, also known as boxer’s fracture, is the most common metacarpal fracture. Percutaneous Kirschner-wire (K-wire) pinning has been shown to produce favorable clinical results. However, the fixation power of K-wires is a major concern. Plate fixation is also a surgical option, but it has the disadvantages of tendon adhesion, requirement of secondary surgery for removal of the implant, and postoperative joint stiffness. A fixation method that causes little soft tissue damage and provides high biomechanical stability is required for patients with fifth metacarpal neck fracture for whom surgical intervention is indicated. The present study proposed fixation using K-wires and a cerclage wire to treat fifth metacarpal neck fracture. The fixation power of this new method was compared with that of K-wires alone and plates.

**Methods:**

We used a saw blade to create transverse metacarpal neck fractures in 16 artificial metacarpal bone specimens, which were then treated with four types of fixation as follows: (1) locking plate with five locking bicortical screws (LP group), (2) regular plate with five bicortical screws (RP group), (3) two K**-**wires (K group), and (4) two K**-**wires and a figure-of-eight cerclage wire (KW group). The specimens were tested by using cantilever bending testing on a material testing system. The stiffness of the four fixation types was determined by observing force–displacement curves. Finally, the Kruskal–Wallis test was adopted to process the data, and the Mann–Whitney exact test was performed to conduct paired comparison between the fixation types.

**Results:**

The fixation strength levels of the four fixation approaches for treating fifth metacarpal neck fracture were ranked in a descending order of LP group (24.6 ± 5.1 N/mm, median ± interquartile range) > RP group (22.2 ± 5.8 N/mm) ≅ KW group (20.1 ± 3.2 N/mm) > K group (16.9 ± 3.0 N/mm).

**Conclusion:**

The fixation strength of two K-wires was significantly higher when reinforcement was provided using a figure-of-eight cerclage wire. The strength of the proposed approach is similar to that of a regular plate with five bicortical screws but weaker than that of a locking plate with the same amount of bicortical screws. Cerclage wire-integrated K-wires can be an alternative method that avoids the excessive soft tissue dissection required for plating in open reduction internal fixation for fifth metacarpal neck fracture.

## Background

Metacarpal fractures are common orthopedic injuries [1] that account for 13% of hand fractures and 23% of forearm fractures [2–4]. The fifth metacarpal neck fracture, also known as boxer’s fracture, is the most common type of metacarpal fracture, constituting 50% of metacarpal fractures [2]. Because of the activity performed by the intrinsic muscles of the hand, patients with fractures in the fifth metacarpal neck are likely to develop volar angulation deformity. If the fracture is treated nonoperatively, volar malunion with dorsal angulation and joint stiffness is common [5, 6]. Therefore, an increasing number of doctors and patients are opting for surgical treatment instead. The surgical indication of fifth metacarpal fracture is generally considered to be fracture angulation of more than 45° resulting in a considerable decrease in grip strength and range of motion [1, 7].

Of the various fixation methods for metacarpal neck fracture, K-wire fixation is less invasive and has been shown to have outcomes superior to those of plating [1, 3]. Intramedullary pinning has been reported to have better outcomes than crossed and transverse pinning because of its minimal violation of soft tissue [8–10]. Despite causing soft tissue invasion, open reduction and internal fixation (ORIF) still has a role in treating patients with multiple concurrent metacarpal fractures, those who need higher fracture biomechanical stability, those who are unable to protect exposed pins, and those who cannot tolerate a period of immobilization [11]. Plates, available in regular and locking forms, are internal fixation devices commonly used for metacarpal neck fractures. They provide high mechanical strength, but many complications have been reported, including metacarpal head avascular necrosis, nonunion, severe tendon irritation, and high rate of stiffness due to excessive soft tissue damage [1, 7, 12–14]. For the minimization of soft tissue damage and reduction of implant profiles in ORIF, we propose a novel fixation method that integrates K-wires with a cerclage wire. This new method may provide higher mechanical stability and avoid the complications that arise from the use of plates.

The purpose of our study was to investigate the degree of strength that a cerclage wire can add to K-wires and to compare the biomechanical stability of this new fixation method with that achieved using two commonly used plate methods.

## Methods

### Specimen preparation

Obtaining an adequate number of real human specimens with identical bone quality and size is difficult, so we used artificial metacarpal bone (3B Scientific GmbH, Hamburg, Germany) in our experiments. A total of 16 artificial metacarpal bone specimens were employed. A metacarpal neck fracture was generated in the specimens using a 0.4-mm saw blade. The fracture distance was 13 mm from the distal articular surface.

### Fixation approaches and stability test

The specimens were assigned to four fixation technique groups, and all fixations were performed by a single senior hand surgeon (Yung-Cheng Chiu).

**Fig. 1:**
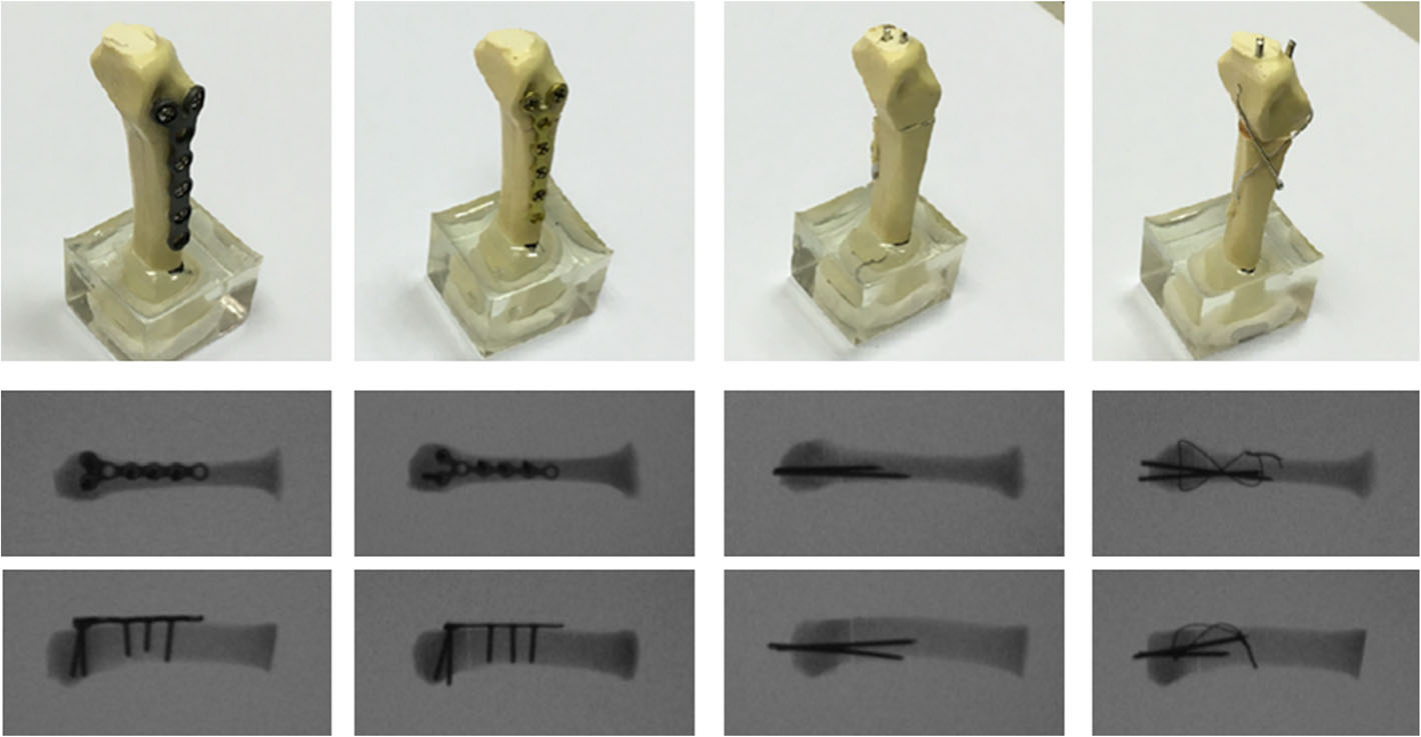
Artificial metacarpal bones and four fixation types of the metacarpal neck fracture. **a** Locking plate with five locking bicortical screws (LP group). **b** Regular plate with five bicortical screws (RP group). **c** Two K-wires (K group). **d** Two K-wires and a figure-of-eight cerclage wire (KW group). Radiographs of the four fixation types in superior and lateral views are also shown

**Fig. 2:**
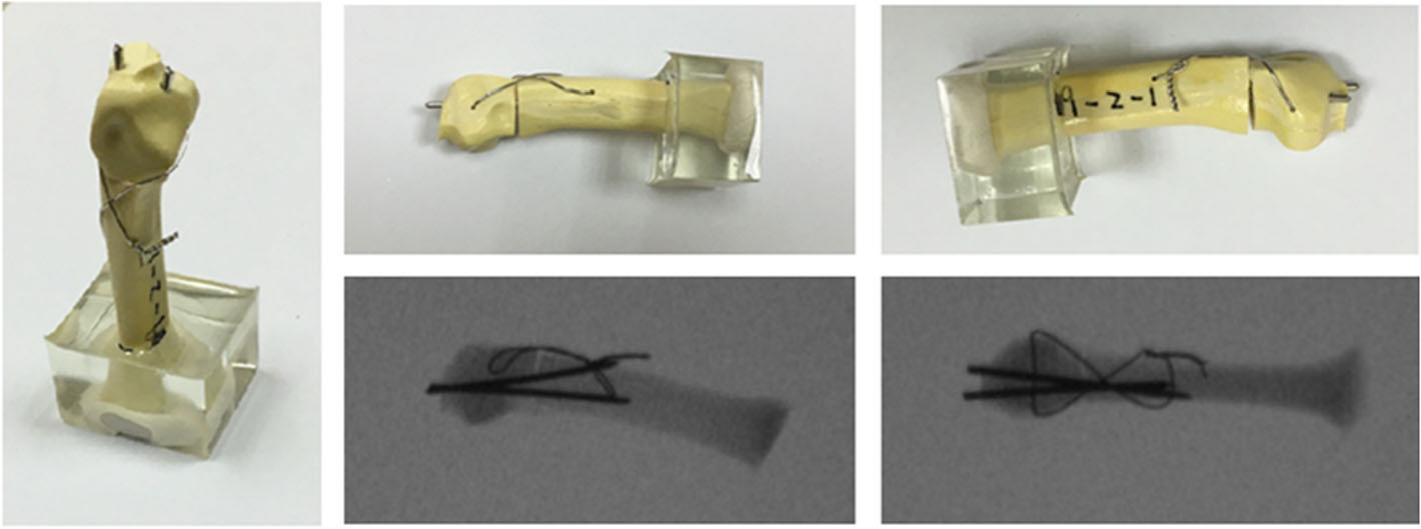
Details of the fixation approach employing two K-wires and a figure-of-eight cerclage wire (KW group)

- Group 1—Locking plate with five locking bicortical screws (LP group). The specimens were fixed using a six-hole Y-shaped locking plate and 2.3-mm-diameter locking screws (Stryker, Germany). The locking plate was fixed at the dorsum of the metacarpal using three bicortical proximal locking screws and two bicortical distal locking screws (Fig. 1a).
- Group 2—Regular plate, nonlocking, with five bicortical screws (RP group). The specimens were fixed using a six-hole Y-shaped nonlocking plate and 2.3-mm-diameter compression screws (Stryker, Germany). The nonlocking plate was fixed at the dorsum of the metacarpal with three bicortical proximal compression screws and two bicortical distal compression screws (Fig. 1b).
- Group 3—Two K-wires (K group). The specimens were stabilized using two 1.5-mm-diameter K-wires inserted from the dorsal medial and lateral sides of the metacarpal head, penetrated the fracture site, and fed out from the proximal volar cortex; fracture reduction was maintained with manual axial compression during the surgery (Fig. 1c).
- Group 4—Two K-wires with figure-of-eight cerclage wire (KW group). The specimens were stabilized using two 1.5-mm-diameter K-wires inserted from the dorsal medial and lateral sides of the metacarpal head, penetrated through the fracture site, and fed out from the proximal volar cortex; fracture reduction was maintained with manual axial compression during the surgery. Subsequently, an 18-gauge needle was mounted on an electric drill to serve as the wire guide. Two wire guides were drilled transversely into the distal and proximal parts of the fractured metacarpal parallel at approximately 0.5 cm from the fracture site. A 25-gauge stainless steel wire was used to pass the two wire guides to shape the wire into a figure of eight at the dorsum of the metacarpal after the two needles were withdrawn (Fig. 1d and Fig. 2).

**Fig. 3:**
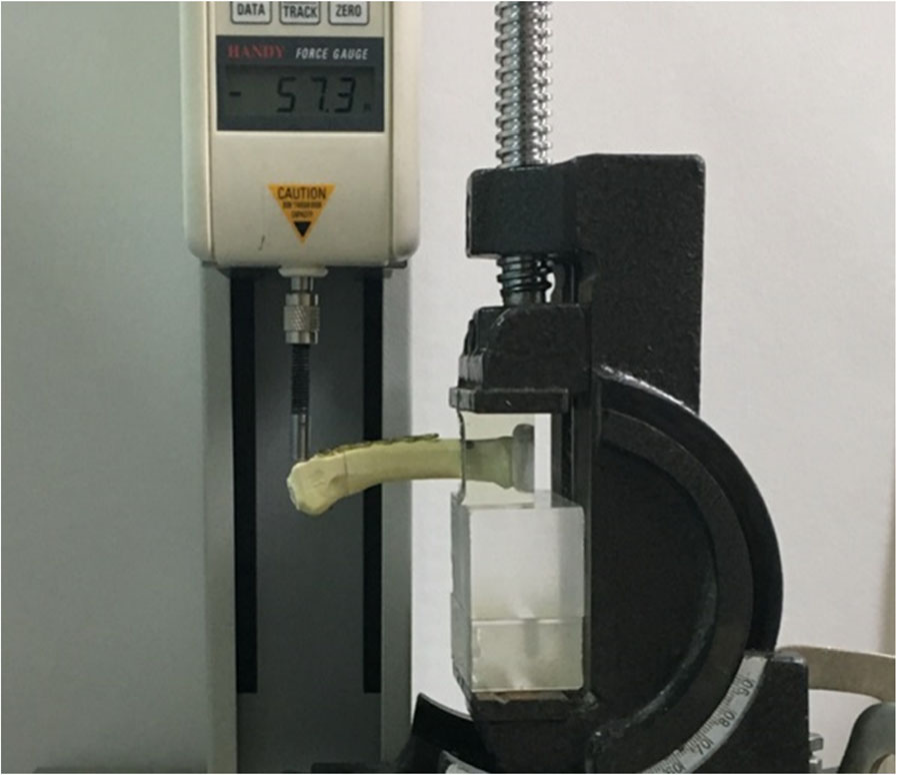
Experimental setup for cantilever bending tests. The specimen has fixation of a locking plate with five locking bicortical screws

Before mechanical testing, the proximal end of each specimen was held in a custom fixture using molded epoxy clamps. Cantilever bending tests were conducted using a material testing system (JSV-H1000, Japan Instrumentation System, Nara, Japan) (Fig. 3). Following preloading to 5 N, a perpendicular load was applied to the dorsal side of the specimen at a distance of 53 mm from the fixture until failure. The crosshead speed was 10 mm/min. The experimental setup was similar to that used in other studies [12, 15]. Force–displacement data were recorded, and the bending stiffness of each specimen was determined.

### Statistical analysis

The stiffness of the specimens with metacarpal neck fracture and four fixation types are summarized as the median value (interquartile range [IQR]). The Kruskal–Wallis test was used to compare the differences between the four fixation types. Post hoc pairwise comparisons were conducted using the Mann–Whitney test. Statistical significance was set at *P* < 0.05. All statistical analyses were performed using SPSS Version 19 (IBM Corporation, Armonk, NY, USA).

## Results

**Fig. 4:**
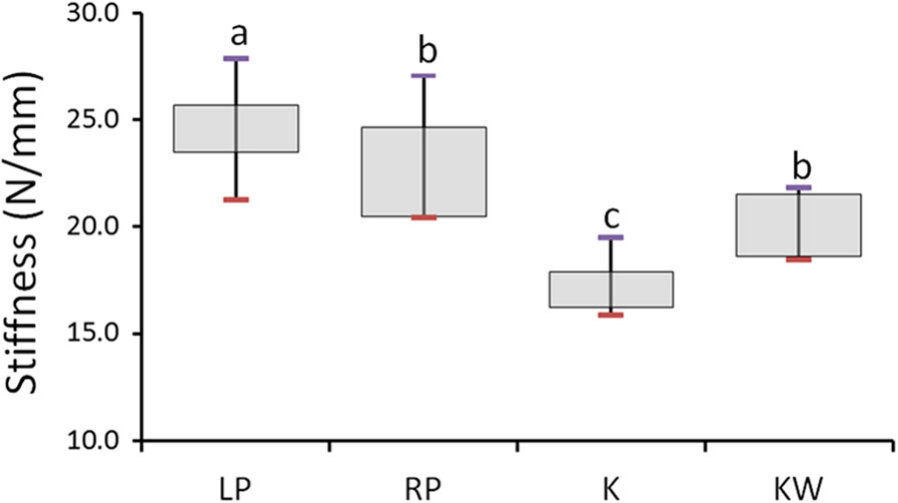
Box plot showing the stiffness of the four fixation types. Post hoc pairwise comparisons were conducted using the Mann–Whitney test; use of the same letter indicates no significant difference at the 0.05 level

The experimental results are presented in Table 1 and Fig. 4. The highest median stiffness was obtained for the LP group specimens and was equal to 24.6 ± 5.1 N/mm. The median of the RP group (22.2 ± 5.8 N/mm) was slightly higher than that of the KW group (20.1 ± 3.2 N/mm), but the difference was nonsignificant. The most unstable fixation type was that in the K group, for which the median stiffness was only 16.9 ± 3.0 N/mm, 15.9 and 23.9% lower than that for the KW and RP groups, respectively. The fixation stiffness of the four types of fixation in fifth metacarpal neck fracture thus followed the order LP group > RP group ≅ KW group > K group.

**Table 1:** Stiffness (unit: N/mm) of the four fixation types used to fix metacarpal neck fracture

Group	Median	IQR	Max	Min	*P*
LP	24.6	5.1	27.9	21.3	0.001
RP	22.2	5.8	27.1	20.4	
K	16.9	3.0	19.5	15.9	
KW	20.1	3.2	21.8	18.5

## Discussion

Fifth metacarpal neck fracture is a common hand fracture. K-wires and plates are the commonly used internal fixation devices; however, numerous complications and high rates of postoperative joint stiffness are reported due to the excessive soft tissue damage that these devices cause [1, 7, 12–14]. Herein, we proposed a mixed fixation of K-wires and a cerclage wire. This fixation approach requires less soft tissue dissection than plate insertion, has mechanical strength comparable to that of a nonlocking plate, and may avoid the complications caused by plate fixation. Additionally, the cost of the proposed approach is considerably lower than that of plate fixation.

In this study, we used artificial metacarpal bones to conduct experiments because of difficulty with acquiring bones from corpses with consistent qualities. Fifth metacarpal neck fracture is often experienced by young people, but obtaining metacarpal bones from the corpses of young people is particularly difficult. Consequently, studies have tended to conduct experiments using porcine or artificial metacarpal bones [12, 16–18]. This study compared the strength of different osteosynthesis techniques for fixation and therefore entailed selecting specimens with almost identical geometrics and material properties. Accordingly, artificial bones were employed because of their uniformity and consistency [19]. According to the American Society for Testing and Materials (ASTM) F-1839-08, “Standard Specification for Rigid Polyurethane Foam for Use as a Standard Material for Testing Orthopaedic Devices and Instruments” states that “the uniformity and consistent properties of rigid polyurethane foam make it an ideal material for comparative testing of bone screws and other medical devices and instruments” [20].

We conducted cantilever bending tests, which differ slightly from the physiological loading tests commonly used in a clinical setting. However, no existent in vitro biomechanical test is able to reflect actual amounts of physiological loading. In addition to the cantilever bending test, previous studies have adopted tests including the three-point bending test [17], modified three-point bending test [13, 21, 22], four-point bending test [16, 18], and torsional test [12, 23]. We employed the cantilever bending test instead of a bending test mainly because the aim of the study was to explore fixation approaches for metacarpal neck fracture not metacarpal shaft fracture. Previously, the maximum fracture force and stiffness were common indexes for scholars to determine fixation strength [12, 14, 16, 17, 21, 23]. In this study, however, the strength was assessed by evaluating the stiffness alone. We assumed that during fracture healing, no matter what fixation is used, refracture of the fifth metacarpal neck as a result of an extremely strong active or passive force does not and should never occur. By contrast, stiffness indicates the structure stiffness of a fixation. Thus, measuring stiffness is more meaningful than measuring the maximum fracture force in clinical practice.

Research has identified that bone plate fixation is the strongest fixation [16, 24–27]. Similarly, the present study determined that the stiffness of the fixation using two K-wires was lower than that using a locking plate and regular plate. Malasitt et al. [13] compared the strength of K-wire and locking plate fixation by conducting experiments on porcine second metacarpals and revealed that the K-wire fixation exhibited higher initial stiffness. The experiments of the present study verified that the stiffness of locking plate fixation (24.61 + 5.12 N/mm) was higher than that of regular plate fixation (22.17 + 5.12 N/mm). Likewise, Ochman et al. [14] implemented modified three-point bending tests to examine pig metacarpal specimens with various types of fixation. The results indicated that when monocortical bone fixation was employed, the stiffness of the fixation using a locking plate (83 + 35 N/mm) was higher than that using a regular plate (46 + 12 N/mm). Doht et al. [21] also conducted modified three-point bending tests on pig metacarpal bones. Despite the mean stiffness of the locking plate group exceeding that of the regular plate group, the difference was nonsignificant.

The stiffness of the KW group was lower than that of the LP group, but no significant difference was discovered between that of the KW and RP groups. Additionally, KW fixation exhibited much greater stiffness than K-wire fixation, indicating that using two K-wires and one figure-of-eight cerclage wire to treat fifth metacarpal neck fracture is reliable.

An increasing amount of evidence is demonstrating that some innovative procedures and modern, fashionable, expensive locking plates do not always achieve superior outcomes to nonsurgical treatment or use of a simple fixation device (e.g., K-wires) [28–30]. Orthopedic and trauma surgeons should consider the possibility of nonsurgical treatment before resorting to an operation. Maffulli indicated that if surgical intervention is inevitable, fixation using devices with a low profile should be prioritized [31]. Pinning fractured bones using K-wires is a reliable approach in treating boxer’s fracture. Compared with plate fixation, K-wire fixation requires less soft tissue dissection [9] and thus results in a lower rate of postoperative extensor tendon adhesion. Nonetheless, K-wire fixation does have some disadvantages, including exposed hardware, a longer period of immobilization, and lower resistance to angulation and rotational deformity force wire migration, and these disadvantages make this method inappropriate for some patients [32, 33]. Requiring much less soft tissue dissection than what is needed in plate fixation, K-wire and figure-of-eight cerclage wire fixation prevents K-wires from migrating, avoids hardware exposure, and has similar strength to plate fixation, resisting deformity forces and thus potentially reducing the postoperative immobilization period. The proposed surgical treatment modality (two K-wires and one figure-of-eight cerclage wire) had lower profile than plates, which may reduce complications caused by using a plate, including tendon irritation, joint stiffness, and metacarpal head ischemia necrosis. Regarding the cost-effectiveness, the costs of locking plate and regular plate fixation at our institution are US$1500 and US$200, respectively, whereas that of KW fixation using two K-wires and one figure-of-eight cerclage wire is only US$5. Therefore, given our aforementioned results and the present discussion, we conclude that KW fixation is a feasible alternative fixation for treating fifth metacarpal neck fracture.

The approach of integrating a figure-of-eight cerclage wire to two K-wires and adopted in the present study was inspired by the use of tension band wiring (TBW) fixation [34] in a previous study to treat patella transverse fracture. TBW is widely employed to treat limb fractures, including distal clavicle fracture [35], olecranon fracture [36], and medial malleolar fracture [37]. The advantages of TBW are (1) higher antirotation force [3] compared with that in K-wire fixation, (2) higher antibending force compared with that in wire fixation, and (3) lower profile compared with the bone plate, which reduces tendon irritation. Therefore, bones heal more effectively when TBW is applied at the tension side of a fracture [38, 39]. In our proposed method, TBW is inserted in the dorsal side of the fractured fifth metacarpal neck because this fixation is mechanically advantageous. Usually, the fractured fifth metacarpal neck is tilted toward the palm because of the traction power of intrinsic muscle, meaning that the dorsal side is the tension side. By using the fixation of two K-wires and one figure-of-eight cerclage wire, the dorsal tension side became the compression side, enabling the dorsal volar cortex (fractured bones) to heal faster.

This study was subject to several limitations. First, to ensure that the specimens used had the same geometry and material properties, we used artificial bones to conduct experiments. Although artificial bones have been used previously [12, 40], they are not able to simulate the actual properties of human bones such as inhomogeneity and anisotropy and do not have the actual structure of trabecular bones. Second, as was the case in most previous in vitro biomechanical experiments, the fracture pattern created was a simple fracture [12, 16–18]. Third, we conducted cantilever bending tests to assess the fixation strength of the distinct fixation types, and such testing does not reflect all actual physiological conditions; in fact, no mechanical testing is able to reflect all actual physiological conditions. Fourth, as previous studies have indicated [18, 19], artificial and animal bones do not contain soft tissues such as muscles, ligaments, and tendons. Although the absolute values measured in this study will hence be different from those under actual conditions, we held that this difference would not influence the ranking of the four fixation types regarding their fixation strength. In the future, we will collect clinical results to evaluate the effect of soft tissue when the two K-wires and a figure-of-eight cerclage wire fixation approach is employed.

## Conclusion

In this study, we proposed a new fixation approach with two K-wires and one figure-of-eight cerclage wire for treating fifth metacarpal neck fracture in patients for whom open reduction internal fixation is indicated. On the basis of the experimental design and limitations, we conclude that the figure-of-eight cerclage wire significantly increases the biomechanical stability of the two K-wires. The fixation strength of the proposed fixation is similar to that of a regular plate with five bicortical screws but weaker than that of a locking plate with five bicortical screws. Integrating a figure-of-eight cerclage wire with K-wires is an alternative fixation method that avoids the excessive soft tissue dissection that occurs in open reduction internal fixation for fifth metacarpal neck fracture.

## Abbreviations

K group: Two K-wire group
KW group: Two K-wires and a figure-of-eight cerclage wire group
LP group: Locking plate with five locking bicortical screw group
RP group: Regular plate with five bicortical screw group
TBW: Tension band wiring
